# Environmental Safety Analysis of Red Mud-Based Cemented Backfill on Groundwater

**DOI:** 10.3390/ijerph18158094

**Published:** 2021-07-30

**Authors:** Shuai Li, Yulin Zhang, Ru Feng, Haoxuan Yu, Jilong Pan, Jiwei Bian

**Affiliations:** School of Resources and Safety Engineering, Central South University, Changsha 410083, China; shuaige@csu.edu.cn (S.L.); 8210183024@csu.edu.cn (Y.Z.); ru-feng@csu.edu.cn (R.F.); bianjiwei@csu.edu.cn (J.B.)

**Keywords:** Bayer process red mud (BRM), red mud-based cemented backfill (RMCB), environmental safety analysis, groundwater environment, fuzzy comprehensive evaluation

## Abstract

As one of the main industrial solid wastes, there are a large number of free alkaloids, chemically bound alkaloids, fluoride, and heavy metal ions in Bayer process red mud (BRM), which are difficult to remove and easily pollute groundwater as a result of open storage. In order to realize the large-scale industrial application of BRM as a backfilling aggregate for underground mining and simultaneously avoid polluting groundwater, the material characteristics of BRM were analyzed through physical, mechanical, and chemical composition tests. The optimum cement–sand ratio and solid mass concentration of the backfilling were obtained based on several mixture proportion tests. According to the results of bleeding, soaking, and toxic leaching experiments, the fuzzy comprehensive evaluation method was used to evaluate the environmental impact of BRM on groundwater. The results show that chemically bound alkaloids that remained in BRM reacted with Ca^2+^ in PO 42.5 cement, slowed down the solidification speed, and reduced the early strength of red mud-based cemented backfill (RMCB). The hydration products in RMCB, such as AFT and C-S-H gel, had significant encapsulation, solidification, and precipitation inhibition effects on contaminants, which could reduce the contents of inorganic contaminants in soaking water by 26.8% to 93.8% and the leaching of toxic heavy metal ions by 57.1% to 73.3%. As shown by the results of the fuzzy comprehensive evaluation, the degree of pollution of the RMCB in bleeding water belonged to a medium grade Ⅲ, while that in the soaking water belonged to a low grade II. The bleeding water was diluted by 50–100 times to reach grade I after flowing into the water sump and could be totally recycled for drilling and backfilling, thus causing negligible effects on the groundwater environment.

## 1. Introduction

As the second most important metal after steel, aluminum and its alloys are widely used in construction, transportation, electrical appliances, machinery, and other industries due to their excellent properties [1]. At present, 95% of the world’s aluminum companies use the Bayer process to treat bauxite ore in order to produce alumina and then to obtain aluminum metal through electrolysis; the main solid waste produced is called Bayer process red mud (BRM). Therefore, BRM is a type of red, silty, and strong alkaline main industrial solid waste generated in the process of alumina industry [2]. Statistically, every ton of electrolytic aluminum produced will discharge 2.0–3.6 tons of BRM. At present, the total output of BRM in the world has reached 90 million t/a, the total amount of surface storage has exceeded 2 billion tons, and the annual production will continue to increase in the next ten years [3].

Traditionally, fresh BRM would be directly discharged and stored in the open-air storage yard without any further treatment [4]. Due to the large amounts of free alkaloids, chemically bound alkaloids, fluoride, and heavy metal ions in BRM, the open surface storage of BRM not only occupies a large amount of land but also easily generates dust and groundwater pollution, and causes ecosystem destruction [5]. The alkaline liquid leached during long-term storage penetrates the ground, polluting water sources and causing soil alkalization [6]. In addition, the exposure of BRM to the air forms dust, which severely pollutes the atmosphere [7]. In 2010, a BRM dam burst in Hungary, and approximately 100,000 cubic meters of highly alkaline BRM leaked, polluting 1017 hectares of agricultural land and making it unsuitable for crops [8]. Taneez [9] analyzed the significant threat to soil and vegetation posed by BRM due to its high alkalinity and fine grain size resulting from the Bayer process. With the implementation of environmental protection policies and the emphasis on sustainable development, the disposal of BRM has become a critical bottleneck restricting the development of the alumina industry, and placing a heavy burden on socio-economic development and environmental protection [10]. Therefore, research on the safe disposal and comprehensive utilization of BRM has become a new research hotspot.

In view of the potential hydration properties of red mud during sintering and combination, it can be used in the production of cement, bricks for building, glass, and special ceramics, as well as in additives or auxiliary materials for asphalt materials, roadbed materials, thermal insulation materials, and other building materials [11,12,13,14]. BRM with a large specific surface area shows excellent adsorption capacity for metal ions and radioactive elements, which can be used as an adsorbent for environmental restoration, such as waste gas treatment, wastewater treatment, and soil remediation [15,16,17]. In addition, BRM is used to produce coal-burning desulfurizers, polymer water-purifying agents, and siliceous calcium agricultural fertilizers [18,19]. However, there are a large number of alkaline compounds (Na_2_O and K_2_O) that are difficult to eliminate, as well as fluorine, aluminum, and many other impurities, which pose an environmental risk in the preparation of building or functional materials [20]. Thus, the contradiction between technology and economy, as well as safety and environmental protection issues, means the comprehensive utilization of BRM is still at the experiment and research stage, limiting its industrial application and large-scale popularity [21]. Currently, the emissions of BRM in China are as high as 600 million tons, while the comprehensive utilization rate is only 4% [22]. Therefore, it is exceptionally urgent to minimize the hazards of BRM and achieve its multichannel and large-scale utilization. 

Cemented backfill mining involves a well-proportioned mixture of solid wastes, cementing materials, and water, which are transported from a surface backfilling station to the underground mining goafs by a pipeline; excellent superiority is shown in ground pressure management after consolidation and hardening [23]. As one of the main industrial solid wastes, phosphogypsum contains large amounts of strong acidic phosphates and sulfates, which are difficult to remove but easily pollute the groundwater. The Kaiyang mine in Guizhou province has used phosphogypsum as a backfilling aggregate for more than ten years, thus demonstrating a safe means of disposal of main industrial solid wastes [24]. Coal gangue and fly ash are two other main industrial solid wastes; the Suncun mine in Shandong province has reused them as backfilling aggregate materials for nearly 20 years, which is not only beneficial in ensuring the safety of mining but also provides a new means of solid waste disposal [25]. Zhu [26] used red mud from the sintering process as a partial replacement of binders in cemented backfill mining practices. However, owing to the unique physicochemical properties of BRM, to date, no studies have been conducted on red mud-based cemented backfill (RMCB) while using such types of BRM [27]. Therefore, it is innovative and meaningful to explore the feasibility of reusing BRM as a backfilling aggregate for the large-scale backfill treatment of mining goafs.

Due to the large number of contaminants in BRM, the open surface storage of BRM may easily generate groundwater and soil pollution, and microorganisms and plants destruction. In order to realize the large-scale industrial application of BRM as a backfilling aggregate for underground mining and simultaneously avoid polluting groundwater, the most important aspect is to prevent the transfer of BRM contaminants from surface storage to the underground goafs. Since there is little soil or microorganisms but lots of hard stones and groundwater in the underground mining goafs, then the main environmental safety problem caused by BRM for backfilling is groundwater pollution. In this paper, the material characteristics of BRM were analyzed through physical, mechanical, and chemical composition tests. The optimum cement–sand ratio and solid mass concentration of the backfilling were obtained based on a large number of mixture proportion tests. Using the results of bleeding, soaking, and toxic leaching experiments, a fuzzy comprehensive evaluation method was used to comprehensively evaluate the environmental impact of BRM on groundwater systems.

## 2. Materials and Methods

**Figure 1:**
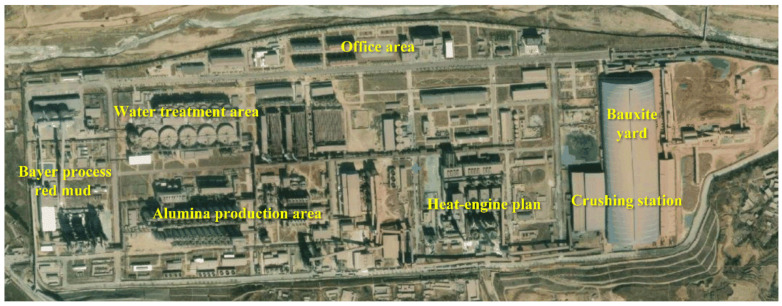
Satellite image of an alumina plant in Shanxi province.

Unlike lateritic bauxite ores in other countries, bauxite ores in China are mainly of the ancient weathering crust type, and nearly 50% are distributed in Shanxi province. Due to the similar minerogenetic conditions and mineral processing methods, BRM in Shanxi province shows similar physical and chemical features [28]. Taking an alumina plant in Shanxi province as an example (see Figure 1), the Bayer process mainly includes the crushing and pulping of bauxite ore, high-pressure digestion of aluminum oxide, separation and washing of red mud, and other production processes. The principle of the Bayer process is dissolving the alumina in bauxite ore with strongly basic NaOH at a high temperature; a sodium aluminate solution and BRM are generated and separated, and then the sodium aluminate decomposes and generates aluminum hydroxide at a low temperature. Finally, the alumina product is obtained after washing and calcining [29]. The Bayer process can be simplified as Equation (1)

Al_2_O_3_ × H_2_O + 2NaOH + aq ⇋ 2NaAl(OH)_4_ + aq (1)

**Figure 2:**
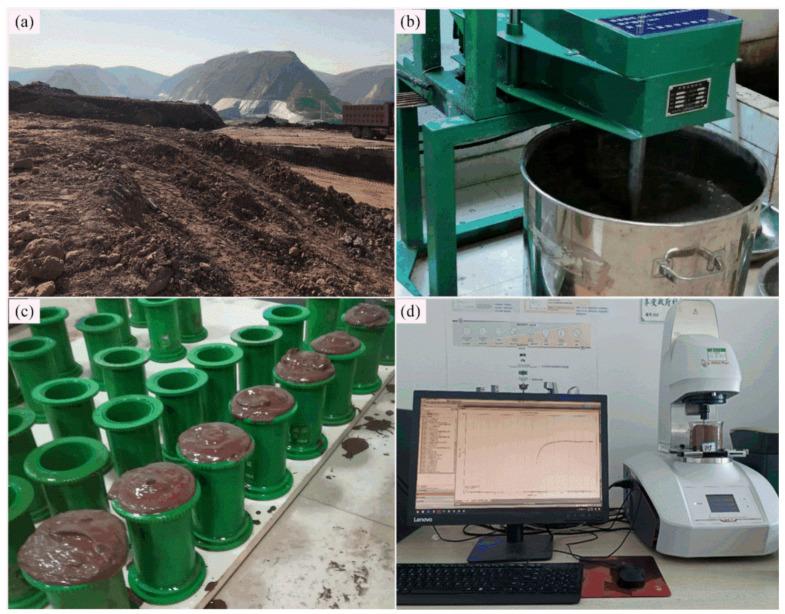
Tests of RMCB: (**a**) dry yard of BRM; (**b**) preparation of RMCB; (**c**) mixture proportion test; (**d**) shear rheological test.

About 2 tons of fresh BRM were packed and transported to the backfilling laboratory of Central South University (see Figure 2). The particle size, oxide content, and mineral composition analysis of BRM were commissioned by Changsha Research Institute of Mining and Metallurgy Co., Ltd. which is located in Changsha City, Hunan Province, China by using a Laser Particle Size Analyzer (Mastersizer 2000), an X-Ray Fluorescence Spectrometer, and an X-Ray Diffractometer, respectively. BRM is similar to ultrafine soil particles with its median particle size of 3.248 μm and a specific surface area of 2940 m^2^/kg, which are much higher than those of ordinary Portland cement 42.5 (PO 42.5) and slag powder. The specific gravity of BRM is 2.424, the plasticity index is 17.0–30.0, and the permeability coefficient is 3.35 × 10^−5^ cm/s. There are large amounts of free alkaloids, chemical binding alkaloids, fluoride, and heavy metal ions in BRM, which are difficult to remove and easily pollute the surface environment as a result of open storage (see Table 1). It can be seen in Table 1 that the main chemical compositions of BRM are Fe_2_O_3_, Al_2_O_3_, SiO_2_, CaO, and MgO. In addition to the major elements, the average concentrations of several hazardous trace elements including As, Pb, Hg, Cd, and Cr were also detected by the ICP-MS analysis. Besides, the main mineral phases in BRM were Perovskite (CaTiO_3_), Hematite (Fe_2_O_3_), Sodium Aluminate (Na_5_AlO_4_), Calcite (CaCO_3_), and Aragonite, etc. [30]. The main chemical binding alkaloid, sodium aluminosilicate hydrate, can be expressed as Equation (2)

xNa_2_[H_2_SiO_4_] + 2NaAl(OH)_4_ → Na_2_O × Al_2_O_3_ × xSiO_2_ × nH_2_O + 2xNaOH (2)

As a solid waste from the smelting industry, S95 slag powder contains large amounts of CaO, SiO_2_, Al_2_O_3_, and other active ingredients, and it is commonly used as a cementitious material in mines [31,32]. About 100 trails of mixture proportion tests were conducted in the backfilling laboratory of Central South University based on an orthogonal experimental design. PO 42.5, S95 slag powder, and lime were selected as cementitious material, cement–sand ratios were set from 1:4 to 1:20, and the solid mass concentration varied from 55% to 65%. By using S95 slag powder as a cementitious material and lime as an activator, RMCB with a cement–sand ratio of 1:6 and a solid mass concentration of 60% solidified quickly within 12 h and reached a high uniaxial compressive strength of 1.1 MPa in 7 days. Therefore, these optimal mixture proportions were selected in order to conduct rheological behavior tests. The results indicated that the collapsing degree of the RMCB was 25 mm and that it showed obvious time-varying shear-thinning characteristics, which made it suitable for the Herschel–Bulkley rheological model [33]. Although the PO 42.5 cement and BRM were evenly mixed and prepared to create a paste state, the RMCB had a slow solidification speed and a very low early strength. Despite the fact that PO 42.5 cement contains a small amount of retarder, the primary reason for slow solidification and low strength is believed to be that chemically bound alkaloids that remain in BRM react with Ca^2+^ in the PO 42.5 cement and produce hydrated garnet, which will greatly reduce the generation of AFT and C-S-H gel as products of the cement hydration reaction [34]. This chemical process can be expressed with Equation (3)

Na_2_O × Al_2_O_3_ × xSiO_2_ × nH_2_O + Ca(OH)_2_ → 3CaO × Al_2_O_3_ nSiO_2_ × (6−2x)H_2_O + NaOH (3)

## 3. Results

**Figure 3:**
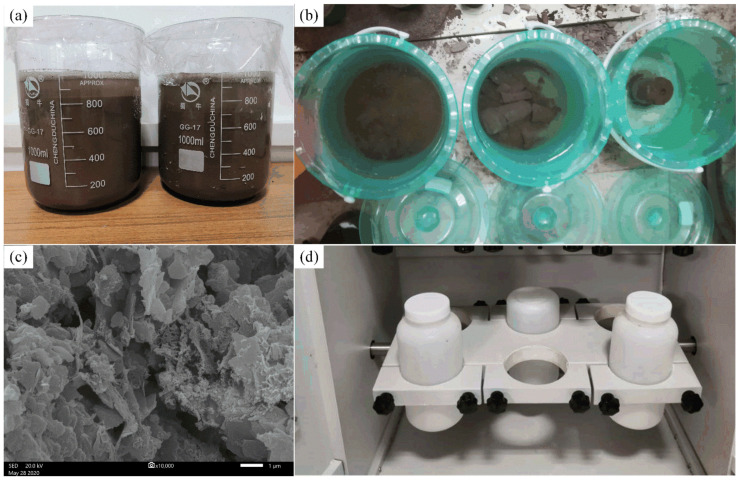
Contamination analysis experiments of RMCB: (**a**) bleeding experiment; (**b**) immersion experiment; (**c**) SEM of RMCB; (**d**) toxic leaching experiment.

Bleeding, immersion, and toxic leaching experiments were conducted to analyze and evaluate the impact of RMCB on groundwater. In addition, the microstructure of RMCB was analyzed through SEM (see Figure 3). The pollutants composition analysis of mine groundwater, bleeding water, and immersing water were commissioned by the Center for Chemical Composition Analysis, Central South University. The pH value, Fluoride, and Chloride were tested by Ion Meters. Meanwhile, the Sulfate, Ammonia nitrogen, Nitrate, and Nitrite were analyzed by different spectral regions of Spectrophotometers. Besides, the heavy metal ions, such as Na, Fe, Cu, Zn, As, Cd, Pb, Cr^6+^, Ni, Ag, Ba, Se, Hg, and Be were detected by the Inductively Coupled Plasma Atomic Emission Spectrometer (Optima 5300 DV).

### 3.1. Bleeding Experiments and Results

**Figure 4:**
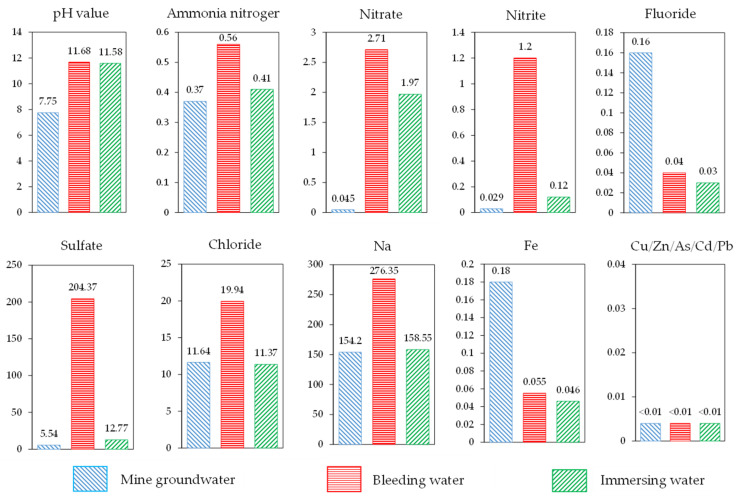
The pollutants composition of mine groundwater, bleeding water, and immersing water.

By using 90% S95 slag powder as a cementitious material and 10% lime as an activator, RMCB with a cement–sand ratio of 1:6 and a solid mass concentration of 60% was prepared and poured into a 1000 mL beaker after being evenly stirred at a high speed. After a period of precipitation, the bleeding water of RMCB was absorbed repeatedly every 10 min and filtered through a 45 μm filter membrane [35]. The pH and concentration of several common heavy metal ions were measured, and fluoride, chloride, nitrate, nitrite, sulfate, ammonia nitrogen, chloride, and other contaminants were detected. The pollutants composition of mine groundwater, bleeding water, and immersing water are shown in Figure 4.

By analyzing the data in Figure 4, the results show that:

(1) Because the RMCB was prepared with alkaline BRM, slag powder, and lime, the pH of the RMCB was about 11.68, which was higher than the value of 7.75 measured in the mine groundwater and slightly lower than the value of 12.1 for fresh BRM.
(2) The concentrations of ammonia nitrogen, nitrate, nitrite, sulfate, chloride, and sodium ions detected in the bleeding water of RMCB were all higher than those in mine groundwater, which is consistent with the phenomenon in which BRM forms white efflorescence on the surface and leads to soil salinization. The primary reason is that a large number of salt agents are added in the Bayer process [36].
(3) The concentrations of fluoride and iron detected in the bleeding water of RMCB were lower than those in the mine groundwater. This is because the AFT and C-S-H gels produced by slag powder and lime generated the effects of inclusion and solidification, thus inhibiting the precipitation of such contaminants [37].
(4) No heavy metal ions, such as copper, zinc, arsenic, cadmium, or lead, were detected in the bleeding water of the RMCB or the mine groundwater. The concentrations of ammonia nitrogen, nitrite, and sodium detected in the bleeding water of RMCB belonged to grade IV of the Groundwater Quality Standard [38], the sulfate concentration belonged to grade III, and the other contaminants all belonged to grade I or grade II.

### 3.2. Immersion Experiments and Results

After consolidation and hardening, RMCB can be used to provide lateral pressure in order to facilitate sliding when unloading rocks, support primitively fractured rocks, and then become a permanent underground support; it is thus inevitably subjected to the leaching and soaking effects of groundwater. Under the long-term effects of groundwater immersion, the toxic dissolution characteristics of RMCB are important indexes for evaluating the environmental safety of the groundwater [39]. Immersion experiments were conducted in a laboratory with the following steps. RMCB with a cement–sand ratio of 1:6 and a solid mass concentration of 60% was placed in a curing box with a constant temperature and humidity for 28 days, and then the RMCB and mine groundwater were sealed in a plastic bucket with a mass ratio of 1:10. After being soaked at room temperature for 30 days, the soaking water of the RMCB was passed through a 45 μm filter membrane. By analyzing the data in Figure 4, the results show that:

(1) Under the long-term effects of groundwater immersion, the pH of the soaking water was about 11.58, which was slightly lower than the values of 11.68 for the bleeding water and 12.1 for the fresh BRM.
(2) The concentrations of ammonia nitrogen, nitrate, nitrite, sulfate, and sodium ions detected in the soaking water of the RMCB were still higher than those in the mine groundwater, which was consistent with the results of the bleeding experiments. The reason was that a large number of salt agents are added in the Bayer process.
(3) The concentrations of fluoride, chloride, and iron detected in the soaking water of the RMCB were lower than those in the mine groundwater, which was also caused by the encapsulation and solidification of the AFT and C-S-H gels. Copper, zinc, arsenic, cadmium, lead, and other heavy metal ions were also not detected in the soaking water of the RMCB and the mine groundwater.
(4) Compared with the results of the bleeding experiments, the concentrations of ammonia nitrogen, nitrate, nitrite, sulfate, chloride, sodium, and iron ions detected in the soaking water decreased by 26.8%, 27.3%, 90.0%, 93.8%, 43.0%, 42.6%, and 16.4%, respectively. Except for the concentrations of ammonia nitrogen, nitrite, and sodium ions, which belonged to grade III of the Groundwater Quality Standard, the other contaminants all belonged to grade I.

### 3.3. Toxic Leaching Experiments and Results

Toxic leaching experiments are important for the identification of solid waste, and the detection results are of great significance in guiding the management and disposal of hazardous solid waste [40]. According to the Chinese National Standard for methods for solid waste leaching and toxic leaching—specifically, the roll-over leaching procedure (GB5096.1-1997) [41]—toxic leaching experiments were carried out on the RMCB to simulate the precipitation of heavy metal ions under extreme conditions, such as creep damage, plastic failure, massive fractures, and overall collapse of the backfill. Well-proportioned RMCB was placed in a curing box with a constant temperature and humidity for 90 days, and then crushed and ground into fine particles. Subsequently, 70.0 g of ground RMCB was placed in a 1 L plastic bottle, and deionized water was added as a leaching agent. The temperature was set at 25 °C the rotation speed was 30 r/min, and the leaching solution was turned over and stirred for 18 h. The leaching bottle was then removed and left standing for half an hour, and the leaching solution was obtained through pressurized filtration with a 0.45 μm microporous membrane. Copper, chromium, lead, zinc, mercury, arsenic, cadmium, nickel, beryllium, silver, selenium, barium, and other metal ion contents were detected, and the results are shown in Table 2.

By analyzing the data in Table 2, the results show that:

(1) In both the fresh BRM or RMCB, the contents of heavy metal ions in the leaching solution were all lower than the standard toxicity limit specified in the Chinese National Standard for the identification of leaching of hazardous solid waste (GB5085.3-2007) [42], indicating that neither the BRM nor the RMCB was a hazardous solid waste.
(2) Small amounts of heavy metal ions, such as Cr^6+^, As, Cu, Zn, Pb, and Ni, were detected in the leaching solution of the BRM, which was consistent with the fact that the content of rare earth elements in BRM in Shanxi province is relatively high.
(3) After adding PO 42.5 cement, only Cr^6+^ and As were detected in the leaching solution of the RMCB, and their detected concentrations were 73.3% and 57.1% lower than those in the BRM, respectively. The SEM images of the RMCB after adding PO 42.5 cement show that the AFT and C-S-H gels generated the effects of inclusion and solidification, thus inhibiting the precipitation of heavy metal ions (see Figure 3c).
(4) After adding slag powder and lime, there were also small amounts of Cr^6+^ and As that were detected in the leaching solution of the RMCB, and their detected concentrations were 20.0% and 33.3% lower, respectively, than those in the RMCB to which PO 42.5 cement was added. This indicated that the hydration products produced by the slag powder and lime were more developed and compact than the PO 42.5 cement, thus showing the more obvious effects of solidification, encapsulation, and inhibition of the precipitation of heavy metal ions.

### 3.4. Environmental Safety Analysis of RMCB on Groundwater

Because contaminants were detected in the bleeding, immersion, and toxic leaching experiments on the fresh BRM and RMCB, the evaluation of the impact of these contaminants on groundwater environments in mining areas must be further studied. In view of the diversity of materials for detection, the inconsistencies in detection methods, and the differences in evaluation indexes, it is necessary to set weights based on the differences in and uncoupling of each contaminant. Thus, the fuzzy comprehensive evaluation method was used to carry out the coupling calculation, fuzzy analysis, and comprehensive evaluation of the water quality results to scientifically evaluate the influence of each contaminant on the groundwater environment [43].

#### 3.4.1. Establishment of the Fuzzy Factor Set and Evaluation Set

A factor set is a set of factors that affect the evaluation of quality, and it is represented by *U* [44]. The pH, ammonia nitrogen, nitrate, nitrite, fluoride, sulfate, chloride, sodium, and iron detected in the bleeding and immersion experiments were selected, and the factor set was *U* = {*u*_1_, *u*_2_,…*u*_9_}. The evaluation set *V* is a set used to evaluate the impacts of various contaminants on the groundwater environment, and the evaluation set was set to *V* = {*v*_1_, *v*_2_, *v*_3_, *v*_4_, *v*_5_} = {I, II, III, IV, V}. The fuzzy comprehensive evaluation of water quality is a comprehensive evaluation of multiple factors [45]. Therefore, the basis of multifactor fuzzy comprehensive evaluation consists of establishing the membership function of a single factor, determining the membership, and obtaining the corresponding membership degree function [46]. In this evaluation, the membership function was obtained by using the distribution curve of a semi-trapezoid function, and the membership degree function of each index was determined according to the content of each index and the quality standard of the groundwater [47,48].

If a contaminant had an extremely low impact on water quality and the water could be used for a variety of purposes, the water quality was defined as grade Ⅰ according to the Chinese Groundwater Quality Standard (GB/T 14848-2017). That is (see Equation (4)), when *j* = 1

(4) $$R_{i1}=\left\{\begin{array}{cc} 1 & C_{i}\leq S_{i1}\\ \frac{S_{i2}-C_{i}}{S_{i2}-S_{i1}} & S_{i1}<C_{i}<S_{i2}\\ 0 & C_{i}\geq S_{i2} \end{array}\right.$$

If the contaminant had a low degree of impact on water quality and the water could be used as centralized drinking water, the quality was defined as grade II. If the contaminant had a moderate impact on water quality and the water could only be used as industrial or agricultural water, the quality was defined as grade III [49]. If the contaminant had a high degree of impact on water quality and the water could only be used as part of the industrial water, the quality was defined as grade IV. That is (see Equation (5)), when *j* = 2, 3, 4

(5) $$R_{ij}=\left\{\begin{array}{cc} \frac{C_{i}-S_{i(j-1)}}{S_{ij}-S_{i(j-1)}} & S_{i(j-1)}\leq C_{i}\leq S_{ij}\\ \frac{S_{i(j+1)}-C_{i}}{S_{i(j+1)}-S_{ij}} & S_{ij}<C_{i}<S_{i(j+1)}\\ 0 & C_{i}\leq S_{i(j-1)}\;\mathrm{or}\;C_{i}\geq S_{i(j+1)} \end{array}\right.$$

If the contaminant had a high degree of impact on water quality and the water had to be treated to reach the standards for use, the quality was defined as grade Ⅴ. That is (see Equation (6)), when *j* = 5

(6) $$R_{i5}=\left\{\begin{array}{cc} 0 & C_{i}\leq S_{i4}\\ \frac{C_{i}-S_{i4}}{S_{i5}-S_{i4}} & S_{i4}<C_{i}<S_{i5}\\ 1 & C_{i}\geq S_{i5} \end{array}\right.$$

where *i* is the pollution factor, *i* = 1, 2, …, 9; *j* is the water quality grade, *j* = 1, 2, 3, 4, 5; *R_ij_* is the membership degree of pollution factor *i* in the water quality of grade *j*; *C_i_* is the measured value of the pollution factor *i*. *S_ij_* is the standard value of the *j*-grade water quality for the pollution factor *i*.

According to this membership degree function, the membership degrees of each evaluation factor of different grades of water quality could be determined so as to ultimately determine an *i* × *j* order fuzzy relational matrix *R* (see Equation (7))

(7) $$R=\left[\begin{array}{cccc} R_{11} & R_{12} & \ldots & R_{15}\\ R_{21} & R_{22} & \ldots & R_{25}\\ \ldots & \ldots & \ldots & \ldots \\ R_{i\;1} & R_{i\;2} & \ldots & R_{i\;5} \end{array}\right]$$

According to Formulas (4)–(7), a fuzzy matrix *R* of order 9 × 5 was constructed, as shown in Equation (8)

(8) $$R=\left[\begin{array}{ccccc} 0 & 0 & 0 & 0 & 1\\ 0 & 0 & 0.94 & 0.06 & 0\\ 0.76 & 0.24 & 0 & 0 & 0\\ 0 & 0 & 0.95 & 0.05 & 0\\ 1 & 0 & 0 & 0 & 0\\ 0 & 0.46 & 0.54 & 0 & 0\\ 1 & 0 & 0 & 0 & 0\\ 0 & 0 & 0.62 & 0.38 & 0\\ 1 & 0 & 0 & 0 & 0 \end{array}\right]$$

#### 3.4.2. Calculation of the Weight Matrix of the Evaluation Factor

Because the contents of each contaminant in the water sample and the impacts on the overall water quality were different, it was necessary to assign a weight to each factor before the comprehensive evaluation [50]. Usually expressed by the matrix *A* = {α_1_, α_2_,…,α_m_}, α_i_ represents the weight coefficient of the *i*-th impact factor among all impact factors, which was calculated according to Equation (9)

(9) $$\alpha_{i}=\frac{C_{i}{/S}_{i}}{\sum_{i}^{n}C_{i}{/S}_{i}}$$

where *C_i_* represents the actual measured value of the *i*-th impact factor, and *S_i_* represents the average value of the *i*-th corresponding standard evaluation values at all levels. The calculations are shown in Table 3.

As shown in Table 3, the weight coefficient matrix of the nine evaluation factors was *A* = {0.2681, 0.1545, 0.0311, 0.1199, 0.0071, 0.1774, 0.0173, 0.2207, 0.0119}. That is, among these factors, the pH value had the greatest influence on the comprehensive evaluation results for water quality, reaching 0.2681, followed by sodium ions and sulfate, which reached 0.2207 and 0.1774, respectively; the content of fluoride had the least influence on water quality—only 0.0071.

#### 3.4.3. Fuzzy Comprehensive Evaluation Results for Water Quality

The mathematical model *B* of the fuzzy comprehensive evaluation of groundwater with RMCB [45,51] can be then expressed as Equation (10)

(10) $$B=AR=(\;a_{1},\;a_{2},\;a_{m})\;\left[\begin{array}{cccc} R_{11} & R_{12} & \ldots & R_{15}\\ R_{21} & R_{22} & \ldots & R_{25}\\ \ldots & \ldots & \ldots & \ldots \\ R_{m1} & R_{m2} & \ldots & R_{m5} \end{array}\right]=(b_{1},b_{2}\ldots ,b_{m})$$

Based on the results of the bleeding experiments on RMCB, the relevant data were substituted into the fuzzy comprehensive evaluation model, and the fuzzy comprehensive evaluation set B of bleeding water quality was calculated with Equation (11)

(11) $$B={\left[\begin{array}{c} 0.2681\\ 0.1545\\ 0.0311\\ 0.1119\\ 0.0071\\ 0.1774\\ 0.0173\\ 0.2207\\ 0.0119 \end{array}\right]}^{T}\left[\begin{array}{ccccc} 0 & 0 & 0 & 0 & 1\\ 0 & 0 & 0.94 & 0.06 & 0\\ 0.76 & 0.24 & 0 & 0 & 0\\ 0 & 0 & 0.95 & 0.05 & 0\\ 1 & 0 & 0 & 0 & 0\\ 0 & 0.46 & 0.54 & 0 & 0\\ 1 & 0 & 0 & 0 & 0\\ 0 & 0 & 0.62 & 0.38 & 0\\ 1 & 0 & 0 & 0 & 0 \end{array}\right]=\{0.0599,\;0.0891,\;0.4825,\;0.0987,\;0.2681\}$$

From the calculation results, it can be concluded that the membership degree of the bleeding water of the RMCB reached the grade III standard, that is, the contaminants in the discharge had a medium influence on the groundwater quality, and the water could be used as industrial and agricultural water. Similarly, the membership degree of the soaking water of the RMCB reached the grade II standard, that is, the pollutants in the soaking water had a low degree of impact on the groundwater quality, and the water could be used as centralized drinking water.

## 4. Discussion

**Figure 5:**
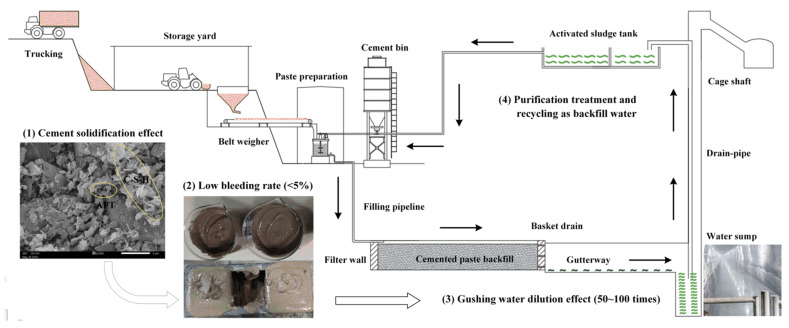
Backfilling technology of RMCB and groundwater recycling process.

As shown in Figure 5 [35], BRM is transported by vehicles and loaded by shovel trucks into a hopper, released at the bottom of the hopper, measured with a belt weigher, and placed into a mixing drum. After mixing with cementitious materials and water at a certain ratio and at a high speed, RMCB is produced and transported via piping through a drill boring and an underground laneway into a mining goaf [52]. As a strongly alkaline solid waste, in BRM, there are large amounts of free alkalis, chemical binding alkali, fluoride, and heavy metal ions, which are difficult to remove and easily pollute the surface environment as a result of open storage [53]. However, with RMCB, the surface pollution caused by BRM will not be transferred underground; the main reasons follow:

(1) A large amount of cementitious material is added to BRM, which will produce abundant hydration products such as AFT and C-S-H gel during the process of the hydration reaction [54]. These hydration products can significantly encapsulate and solidify harmful substances in the BRM and inhibit their precipitation [55]. The toxic leaching results show that the leaching concentrations of chromium, selenium, fluorine, arsenic, lead, and vanadium in RMCB could be reduced by more than 70% compared with those in fresh BRM.
(2) To improve the backfilling effect, RMCB is prepared as a paste with a solid mass concentration of 60% and a bleeding rate of 4%. Less than 0.1 t/h of drainage water, which is only 1% of the normal water inflow in a mine, will be secreted through the pre-buried drainage hole in the filter wall.
(3) After entering the water sump through the gutterway, the bleeding water of the RMCB will be mixed with gushing water caused by underground mining in order to engender a dilution effect. Due to the wave of the inflow of underground water, the dilution ratio can reach at least 50–100 times. The water quality after dilution can reach grade I, and the pH and concentrations of ammonia nitrogen, nitrite, and sodium can be reduced by more than 99%.
(4) The water gushing into the water sump will be discharged into a surface sewage tank for centralized purification, where it will be reused as backfilling water or underground production water to achieve the goal of zero emissions [56].

In summary, under the actions of encapsulation, solidification, and inhibition of precipitation from cementitious materials, the amount of bleeding water from RMCB paste is very small, and the leaching concentrations of harmful substances are greatly reduced. After flowing into the water sump through the gutterway, this water can be further diluted by at least 50–100 times to reach the grade I groundwater standard, and it is finally discharged into a surface sewage tank for centralized purification and recycling, thus realizing the goal of zero emissions.

## 5. Conclusions

This study experimentally examined the possibility of utilizing BRM as a backfilling aggregate. Mixture proportion tests and bleeding, soaking, and toxic leaching experiments were conducted to evaluate the environmental effects of RMCB on a groundwater system. The following conclusions were drawn.

(1) As one of the main industrial solid wastes, there are large amounts of NaOH and chemically bound alkaloids that remain in BRM and are difficult to remove. These components react with Ca^2+^ in PO 42.5 cement and form hydrated garnet, resulting in slow solidification speed and low early strength of RMCB. After using a lower-cost industrial solid waste, S95 slag powder, as a cementing material and lime as an activator, RMCB with a cement–sand ratio of 1:6 and a solid mass concentration of 60% initially solidified within 12 h, and the 7 d compressive strength could reach 1.1 MPa.
(2) The concentrations of ammonia nitrogen, nitrite, and sodium detected in the bleeding water of the RMCB slurry belonged to grade IV of the Groundwater Quality Standard, and the sulfate concentration belonged to grade III. Because only a few Cr^6+^ and As were detected, the RMCB was obviously not hazardous solid waste, and the concentrations of Cr^6+^ and As were lower than those in fresh BRM by 73.3% and 57.1%, respectively.
(3) The fuzzy comprehensive evaluation method was used to carry out the coupling calculation, fuzzy analysis, and comprehensive evaluation of the water quality with respect to RMCB. The results show that the membership degree of the bleeding water of the RMCB reached the grade III standard, that is, the discharged contaminants had a medium influence on the groundwater quality, and the water could be used as industrial and agricultural water. Similarly, the membership degree of the soaking water of the RMCB reached the grade II standard, that is, the pollutants in the soaking water had a low degree of impact on the groundwater quality, and the water could be used as centralized drinking water.
(4) The SEM images of the RMCB showed that a large number of hydration products, such as AFT and C-S-H gel, were generated during the hydration reaction. Under the actions of encapsulation, solidification, and inhibition of precipitation resulting from these hydration products, the bleeding rate of the RMCB paste was only 4%, and the leaching concentrations of harmful substances could be reduced by more than 70%. After flowing into the water sump through the gutterway, the bleeding water can be further diluted by at least 50–100 times to reach the grade I standard, and can finally be discharged into a surface sewage tank for centralized purification and recycling.

## Figures and Tables

**Table 1:** Chemical composition of different backfilling materials.

Composition (%)	CaO	SiO_2_	Fe_2_O_3_	Al_2_O_3_	MgO	SO_3_	Loss on Ignition
BRM	2.57	19	34.5	23	0.2	-	-
PO 42.5	63.20	20.9	2.77	5.45	2.7	2.54	-
Slag powder	37.85	29.55	0.65	15.22	4.97	2.01	1.17

**Table 2:** Content of heavy metal elements in toxic leaching solution (unit: mg/L).

Metal Element	Cr^6+^	As	Cu	Zn	Pb	Ni	Ag	Ba	Se	Cd	Hg	Be
GB5085.3-2007	5	5	100	5	100	5	5	100	1	1	0.1	0.02
BRM	0.131	0.007	0.03	0.02	0.01	0.01	<0.01	<0.01	<0.01	<0.001	<0.0001	<0.001
RMCB (PO42.5)	0.035	0.003	<0.01	<0.01	<0.001	<0.001	<0.01	<0.01	<0.01	<0.001	<0.0001	<0.001
RMCB (Slag)	0.028	0.002	<0.01	<0.01	<0.001	<0.001	<0.01	<0.01	<0.01	<0.001	<0.0001	<0.001

**Table 3:** Calculation table of weight coefficient of each impact factor.

Factor	*C_i_*	*S_i_*	*C_i_/S_i_*	$\sum_{i}^{n}C_{i}/S_{i}$	*α_i_*
pH value	11.680	8.700	1.343	5.008	0.2681
Ammonia nitrogen	0.560	0.724	0.773		0.1545
Nitrate	2.710	17.400	0.156		0.0311
Nitrite	1.200	2.142	0.560		0.1119
Fluoride	<0.050	1.400	0.035		0.0071
Sulfate	204.370	230.000	0.889		0.1774
Chloride	19.940	230.000	0.087		0.0173
Na	276.350	250.000	1.105		0.2207
Fe	0.055	0.920	0.060		0.0119

## Data Availability

The data presented in this study are available on request from the corresponding author.
